# MW-ACGAN: Generating Multiscale High-Resolution SAR Images for Ship Detection

**DOI:** 10.3390/s20226673

**Published:** 2020-11-21

**Authors:** Lichuan Zou, Hong Zhang, Chao Wang, Fan Wu, Feng Gu

**Affiliations:** 1 Key Laboratory of Digital Earth Science, Aerospace information Research Institute, Chinese Academy of Sciences, Beijing 100094, China; zoulichuan20@mails.ucas.ac.cn (L.Z.); wangchao@radi.ac.cn (C.W.); wufan@radi.ac.cn (F.W.); gufeng@radi.ac.cn (F.G.); 2 College of Resources and Environment, University of Chinese Academy of Sciences, Beijing 100049, China

**Keywords:** high-resolution SAR, Ship detection, MW-ACGAN, Yolo v3

## Abstract

In high-resolution Synthetic Aperture Radar (SAR) ship detection, the number of SAR samples seriously affects the performance of the algorithms based on deep learning. In this paper, aiming at the application requirements of high-resolution ship detection in small samples, a high-resolution SAR ship detection method combining an improved sample generation network, Multiscale Wasserstein Auxiliary Classifier Generative Adversarial Networks (MW-ACGAN) and the Yolo v3 network is proposed. Firstly, the multi-scale Wasserstein distance and gradient penalty loss are used to improve the original Auxiliary Classifier Generative Adversarial Networks (ACGAN), so that the improved network can stably generate high-resolution SAR ship images. Secondly, the multi-scale loss term is added to the network, so the multi-scale image output layers are added, and multi-scale SAR ship images can be generated. Then, the original ship data set and the generated data are combined into a composite data set to train the Yolo v3 target detection network, so as to solve the problem of low detection accuracy under small sample data set. The experimental results of Gaofen-3 (GF-3) 3 m SAR data show that the MW-ACGAN network can generate multi-scale and multi-class ship slices, and the confidence level of ResNet18 is higher than that of ACGAN network, with an average score of 0.91. The detection results of Yolo v3 network model show that the detection accuracy trained by the composite data set is as high as 94%, which is far better than that trained only by the original SAR data set. These results show that our method can make the best use of the original data set, improve the accuracy of ship detection.

## 1. Introduction

Ship detection is an important maritime management technology, including the investigation of illegal fishing areas, oil spill detection, maritime traffic management, and national defense [1,2,3,4,5,6,7,8,9]. Synthetic aperture radar (SAR) is an active microwave imaging sensor whose all-day and all-weather working capacity give it an important place in marine exploration [4,5,6,7,8,9].

With the rise of artificial intelligence, deep learning provides powerful power for SAR ship detection and classification [10,11,12,13,14,15,16,17,18,19,20,21,22,23,24]. Deep learning, with its advantages of high accuracy, fast speed and less human intervention, has gradually become the dominant position in the field of SAR ship detection and classification [10,11,12,13,14,15,16,17,18,19,20,21,22,23,24]. Research has shown that deep learning detection algorithms with stronger generalization capabilities require more patterns of labeled data as support. However, because SAR images are expensive to obtain and difficult to interpret, it is an expensive and time-consuming task to create big data sets of SAR images with different scales. The biggest weakness of lacking SAR ship learning data is bound to cause the insufficiency of the above advantages, because deep learning always needs much labeled data to enrich the learning experience [11]. Some scholars, e.g., Wang et al. [11], Li et al. [24], Sun et al. [25] and Wei et al. [26], released their SAR ship detection datasets, but high resolution SAR ship labeled data sets are still lacking. With the development of SAR imaging technology to high resolution, there is an urgent need for a large number of free and public SAR data sets to adapt to high-resolution SAR ship detection.

SAR image simulation is the main method for overcoming the lack of high-resolution SAR data [27]. Traditional SAR image simulation tools are mainly divided into two categories: signal simulation and image simulation. Signal-based simulation is mostly based on electromagnetic theory to calculate the original radar echo signal and simulate microwave scattering processes, such as the Kirchhoff physical optics method [28], geometric optics approximation [29], integral equation method [30] and Phong model [31]. Image simulation algorithms are mostly based on geometric calculation, using gridding algorithms and ray tracing to approximate the electromagnetic propagation process. Such methods include the grid simulation software SARViz [32], and the ray tracing simulation software RaySAR [33] and CohRaS [34]. However, the calculation processes for these methods are complex, consume much computational memory, and cost a large amount of resources [35]. Moreover, these methods lack real data information in the simulation process, resulting in generated images with insufficient realism.

In recent years, the generative adversarial network (GAN) has achieved outstanding performance in computer vision [36,37,38,39,40,41]. The image generation algorithm based on GAN can generate realistic images from real image data, with low loss and end-to-end advantages. It is widely used in image generation [36,37,38], style transfer [39], super-resolution reconstruction [40,41] and other fields. Many researchers have noticed that the GAN can solve the problem of insufficient SAR data. Ren [27] et al. added the multi-scale structural similarity(MS-SSIM) loss item to the original Auxiliary Classifier Generative Adversarial Networks (ACGAN) loss function proposed by Augustus Odena [42] to generate 28 × 28 SAR land-target objects. Jiayi Guo added an angular loss item to the original GAN network loss function to generate 64 × 64 SAR tank targets with different angles [43]. Schwegmann used the Information Maximizing Generative Adversarial Network (InfoGAN) to generate low-resolution SAR ship targets [44]. Marmanis et al. used the Boundary Equilibrium Generative Adversarial Network (BEGAN) to generate SAR building targets of size 160 × 160 [45]. However, although these works propose the use of the GAN to generate SAR target objects, these methods have some shortcomings. When the network generates high-resolution images, training collapse often occurs due to unstable network gradient updates. It is necessary to constantly adjust parameters to find the optimal solution for generating high-resolution images. At the same time, the ACGAN does not impose constraints on the generated images of different scales. To generate images of different scales, the network structure needs to be changed constantly, which greatly increases the consumption of computing resources. In addition, the generated SAR images are only qualitatively evaluated visually, its practicability is not evaluated in scenarios.

Therefore, we need to conduct in-depth research on two issues. The first issue is how to use GANs to generate high-resolution SAR images of multiple scales stably to solve the bottleneck problem of insufficient SAR data. The second is whether the SAR images generated by GANs can be used in target detection frameworks to improve the accuracy of target detection.

Based on the above, this paper proposes an integrated framework of sample generation and detection in the case of small samples of high-resolution SAR ships. Firstly, an improved ACGAN method called Multiscale Wasserstein Auxiliary Classifier Generative Adversarial Networks (MW-ACGAN) is proposed, which uses the multi-scale Wasserstein distance and gradient penalty to make the network more stable, thus generating multiscale high-resolution SAR ship images. Then, the generated images are combined with the original small samples to train the Yolo v3 model to achieve high-precision ship detection under small samples. The remaining chapters of this paper are arranged as follows: the second section mainly introduces the proposed method, and the third section includes the experiments and results. The fourth part is a summary.

## 2. Method

**Figure 1:**
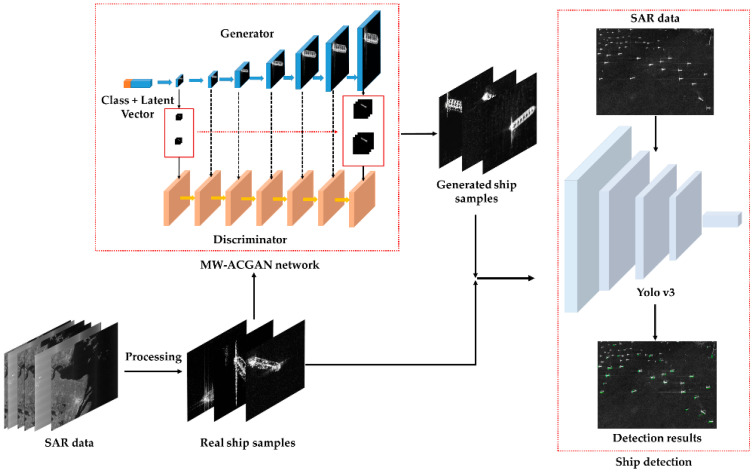
Research flow chart.

Figure 1 shows the flow chart of this study, mainly including two processes. The first part is to generate high-resolution SAR ship images using the MW-ACGAN network. The MW-ACGAN backbone network consists of two parts, one is a generator and the other is a discriminator. The generator obtains images by random vector sampling and bilinear interpolation. The main purpose of the discriminator is to distinguish whether the image comes from the generated image or the real image. The multi-scale loss term is used to transfer the ship image generated by each scale to the discriminator of the corresponding scale, so as to output high-resolution ship images of different scales in real time. The second part is to train the Yolo v3 model by combining the generated high-resolution SAR ship data with the real SAR ship data. Then, some SAR images are used to evaluate the detection performance of the trained Yolo v3 model.

### 2.1. MW-ACGAN Network

#### 2.1.1. GAN and ACGAN

Goodfellow [46] et al. proposed the GAN network, using two kinds of network structure model, generator and discriminator. The generator is used to simulate the data distribution of the real image, and the discriminator is used to judge whether the image is a generated image or a real image. Compared with the image generation model based on maximum likelihood function theory, GAN uses a neural network to simulate more complex functions, and generates higher dimensional images.

The purpose of the generator is to fit the distribution of the real sample data, while the purpose of the discriminator is to distinguish the generated sample from the real sample. The generator G takes the random noise vector z as input and finally outputs the image $X_{\mathrm{fake}}=\;G(z)$. The purpose of the generator is to make the generated distribution $X_{\mathrm{fake}}$ fit the real data distribution $X_{\mathrm{real}}$. The discriminator D inputs the generated image and the real image, and the output probability distribution of the image source P(S|X) = D(X). The purpose of the discriminator is to distinguish between the generated image and the real image. The final generator and discriminator are opponents of the “game,” which includes the minmax adversarial training method and alternate training at the same time. The optimization function is as follows,

(1) $$\underset{G}{\min}\underset{D}{\max}V\left(D,G\right){\;=\;E}_{{x\sim X}_{\mathrm{real}}\left(x\right)}\left[\mathrm{logD}\left(x\right)\right]{\;+\;E}_{{z\sim X}_{\mathrm{fake}}\left(z\right)}[\log\left(1\;-D\left(G\left(z\right)\right)\right)]$$

The optimization objective function is mainly divided into two parts: the optimization for generator G and the optimization for discriminator D. The optimization of discriminator D is shown as,

(2) $$\begin{array}{c} D^{*}\;=\;\arg\underset{D}{\max}V\left(D,G\right)\\ =\;\arg\underset{D}{\max}\left(E_{{x\sim X}_{\mathrm{real}}}\left[\mathrm{logD}\left(x\right)\right]{\;+\;E}_{{x\sim X}_{\mathrm{fake}}}\left[\log\left(1\;-\;D\left(x\right)\right)\right]\right)\\ =\arg\underset{D}{\max}\left(\int X_{\mathrm{real}}\left(x\right)\mathrm{logD}\left(x\right)\mathrm{dx}\;+\;\int X_{\mathrm{fake}}\left(x\right)\log\left(1-D\left(x\right)\right)\mathrm{dx}\right)\\ =\arg\underset{D}{\max}(\int {[X}_{\mathrm{real}}\left(x\right)\mathrm{logD}\left(x\right){\;+\;X}_{\mathrm{fake}}\left(x\right)\log(1-D(x))]\mathrm{dx}) \end{array}$$

The differential equation is obtained,

(3) $$D^{*}\left(x\right)=\frac{X_{\mathrm{real}}\left(x\right)}{X_{\mathrm{real}}\left(x\right){\;+\;X}_{\mathrm{fake}}\left(x\right)}$$

The optimal formula of discriminator D is obtained,

(4) $$\begin{array}{c} \underset{D}{\max}V\left(G,D\right)\;=\;V\left({G,D}^{*}\right)\\ {=E}_{{x\sim P}_{\mathrm{data}}}\left[\log\frac{X_{\mathrm{real}}\left(x\right)}{X_{\mathrm{real}}\left(x\right){\;+\;X}_{\mathrm{fake}}\left(x\right)}\right]{\;+\;E}_{{x\sim P}_{G}}\left[\frac{X_{\mathrm{real}}\left(x\right)}{X_{\mathrm{real}}\left(x\right){\;+\;X}_{\mathrm{fake}}\left(x\right)}\right]\\ =\int X_{\mathrm{real}}\left(x\right)\log\left(\frac{X_{\mathrm{real}}\left(x\right)}{X_{\mathrm{real}}\left(x\right){\;+\;X}_{\mathrm{real}}\left(x\right)}\right)\mathrm{dx}\;+\;\int X_{\mathrm{fake}}\left(x\right)\log\left(\frac{X_{real}\left(x\right)}{X_{\mathrm{real}}\left(x\right){\;+\;X}_{\mathrm{fake}}\left(x\right)}\right)\mathrm{dx}\\ =-2\log2\;+\;\int X_{\mathrm{real}}\left(x\right)\log\frac{X_{\mathrm{real}}\left(x\right)}{{(X}_{\mathrm{real}}\left(x\right){\;+\;X}_{\mathrm{fake}}(x))/2}\mathrm{dx}\\ {=-2\log2\;+\;\mathrm{KL}(X}_{real}\left|\right|\frac{X_{\mathrm{real}}{\;+\;X}_{\mathrm{fake}}}{2}{)\;+\;\mathrm{KL}(X}_{\mathrm{real}}\left|\right|\frac{X_{\mathrm{real}}{\;+\;X}_{\mathrm{fake}}}{2})\\ {=-2\log2\;+\;2\mathrm{JSD}(X}_{\mathrm{real}}{||X}_{\mathrm{fake}}) \end{array}$$

From (4), the optimization goal of GAN discriminator D is to measure the JS divergence between the distribution $X_{\mathrm{real}}$ of real sample data and the distribution $X_{\mathrm{fake}}$ of generated sample data. An optimization for generator G is shown as,

(5) $$G^{*}=\arg\underset{G}{\min}\underset{D}{\max}V\left(G,D\right)$$

From (5), the optimization goal of GAN generator G is to reduce the JS divergence between the distribution $X_{\mathrm{fake}}$ of generated sample data and the distribution $X_{\mathrm{real}}$ of real sample data.

However, the original GAN has difficulty generating a multicategory and high-quality data set [47]. The ACGAN proposed by Odena [42] realized the generation of 128 × 128 high-resolution images by adding the discrimination structure of image categories. The ACGAN adds a conditional category vector c based on the input noise z to constrain the generator and then uses an auxiliary classifier to provide the GAN with the ability to generate high-quality images of multiple categories. Due to the addition of the category discrimination layer, the final objective function contains two parts: the true and false discrimination probability $L_{s}$ and the category discrimination probability $L_{c}$.

(6) $$L_{S}\;=\;E\left[\mathrm{logP}\left({S=\mathrm{real}|X}_{\mathrm{real}}\right)\right]{\;+\;E[\mathrm{logP}(S=\mathrm{fake}|X}_{\mathrm{fake}})]$$

(7) $$L_{C}\;=\;E\left[\mathrm{logP}\left({C=c|X}_{\mathrm{real}}\right)\right]{\;+\;E[\mathrm{logP}(C=c|X}_{\mathrm{fake}})]$$

The optimization goal of the discriminator D is to maximize $L_{S}{\;+\;L}_{C}$, and the optimization goal of the generator G is to maximize $L_{S}{-L}_{C}$. After continuous iteration, the ACGAN can generate high-quality images in multiple categories. However, when the ACGAN generates images with different resolutions, it is often necessary to change the network structure by terminating training, which greatly increases the consumption of computing resources. At the same time, when generating high-resolution images (such as 256 × 256 images), the problems of unstable gradient updates and mode collapses often occur due to the use of the JS divergence to measure data distribution differences. To solve the above problems, we design the following network structure.

#### 2.1.2. MW-ACGAN

**Figure 2:**
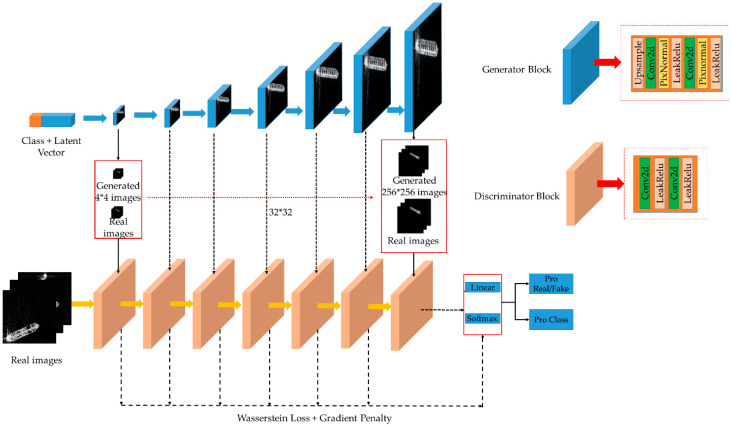
Our proposed network structure.

The proposed MW-ACGAN network structure is shown in Figure 2.

Figure 2 shows the detailed architecture of the proposed MW-ACGAN network. For the generator, as the blue block unit in the upper right of Figure 2, we use an upsampling layer, two convolutional layers, two local response normalization layers and two LeakyReLU activation functions. For the discriminator, we use two convolutional layers and two LeakyReLU activation functions to form a unit module.

We have made two improvements to the original ACGAN network, one is to use Wasserstein distance and gradient penalty terms to make training more stable. The other is to add multi-scale loss term to generate high-quality images of different scales. These two improvements are detailed below. 

##### Wasserstein Distance and Gradient Penalty Terms

The ACGAN uses JS divergence to measure the differences between data distributions,

(8) $${\mathrm{JS}(X}_{\mathrm{real}}||X_{\mathrm{fake}})=\;\frac{1}{2}{\mathrm{KL}(X}_{\mathrm{real}}\left|\right|\frac{X_{\mathrm{real}}{\;+\;X}_{\mathrm{fake}}}{2})\;+\;\frac{1}{2}{\mathrm{KL}(X}_{\mathrm{real}}\left|\right|\frac{X_{\mathrm{real}}{\;+\;X}_{\mathrm{fake}}}{2})\;$$

where, $X_{\mathrm{real}}$ is the probability distribution of real data, and $X_{\mathrm{fake}}$ is the probability distribution of generated data. When JS divergence is used to measure the difference of data distribution, the following problems occur: minimizing the generator loss is equivalent to reducing the JS divergence of $P_{r}$ and $P_{g}$, and in a high-dimensional space, the real data distribution and the generated data distribution usually do not coincide. Therefore, the JS divergence metrics are log2, which causes the gradient of the generator to be 0. Therefore, when the discriminator is overtrained, the generator tends to make the gradient disappear, which makes it difficult to decrease the loss of the generator. When the discriminator is undertrained, the generator will experience unstable gradient updates.

When the Wasserstein distance is used to measure the difference between data distributions, it is defined as follows,

(9) $$W\left(X_{\mathrm{real}}{,\;X}_{\mathrm{fake}}\right)\;=\;\underset{\gamma \sim \prod {(X}_{\mathrm{real}}{,X}_{\mathrm{fake}})}{\inf}E_{\left(x,y\right)}\left[||x\;-y||\right]$$

Even if the two distributions do not overlap in high-dimensional space, the Wasserstein distance can still measure the difference between them so that the problem of the disappearance of the gradient can be solved. However, because the $\underset{\gamma \sim \prod {(P}_{r}{,P}_{g})}{\inf}$ method in (9) can not be solved directly, the Lipschitz transformation is used to transform the formula:

(10) $$W\left(X_{\mathrm{real}}{,\;X}_{\mathrm{fake}}\right)=\frac{1}{K}\underset{{||f||}_{L}\leq K}{\sup}E_{{x\;\sim \;X}_{\mathrm{real}}}\left[f(x)\right]-E_{{x\;\sim \;X}_{\mathrm{fake}}}\left[f(x)\right]$$

In this case, ω is used to define a series of possible functions $f_{w}\left(x\right)$. Then, (10) can be solved by

(11) $$K\;W\left(X_{\mathrm{real}}{,\;X}_{\mathrm{fake}}\right)=\;\underset{{w::|f}_{w}{|}_{L}\leq K}{\max}E_{{x\;\sim X}_{\mathrm{real}}}\left[f_{w}\left(x\right)\right]-E_{{x\;\sim X}_{\mathrm{fake}}}\left[f_{w}\left(x\right)\right]$$

A parameter W neural network $f_{w}$ is constructed to maximize (12). At this time, L is approximately the Wasserstein distance between the real sample data and the generated sample data.

(12) $${L=E}_{{x\;\sim X}_{\mathrm{real}}}\left[f_{w}\left(x\right)\right]-E_{{x\;\sim X}_{\mathrm{fake}}}\left[f_{w}\left(x\right)\right]$$

Finally, the gradient penalty term is added, because the gradient loss term can effectively limit the gradient update range and prevent gradient explosion. The final loss function is as follows:

(13) $$L_{S}^{\prime }=E(||D(X_{S}|S=\mathrm{real})-D(X_{S}|S=\mathrm{fake})||)+\lambda \cdot [{\left({||\nabla_{\hat{z}}D(\hat{z})||}_{2}-1\right)}^{2}]$$

In (13), the first term is the Wasserstein loss, and the second term is the gradient penalty term. The penalty coefficient λ is set to 10. The optimization goal of the final discriminator is to maximize $L^{\prime }{}_{S}{\;+\;L}_{C}$, and the optimization goal of the generator is to maximize $L^{\prime }{}_{S}{-L}_{C}$.

##### Multiscale Loss

Different scenarios have different resolution requirements, so it is an urgent need for a generation network that can generate multiple scales of SAR images at the same time to solve the problem of insufficient data. However, the original ACGAN did not impose constraints on the generation of images at different scales in the generation process, which made it difficult to adapt to the simultaneous generation of multiscale images. To generate multiple-scale images, the network structure needs to be changed and retrained, which greatly increases the amount computing resources consumed.

To solve this problem, we first establish a multiscale data set and downsample the real image size to 256 × 256 at different resolutions. Second, for the generator, we add a multiscale image output layer, that is, use 1 × 1 size convolution to perform channel compression on feature maps of different sizes (such as 4 × 4, 8 × 8, etc.), and then compress them to 3-channel feature maps at the corresponding scale. Next, we generate a 3-channel image and output it to the corresponding structural level discriminator, as shown by the black dotted line in the middle of Figure 2. Finally, we add a multiscale loss term to constrain the network to generate high-quality images with different scales. That is, we add Wasserstein loss and gradient penalty terms of multiple scales, update $\theta_{G}$ and $\theta_{D}$ through back propagation and add the losses of different scales.

(14) $$L^{\prime }{}_{S\_\mathrm{totall}}{\;=\;L}_{S\_4\times 4}^{\prime }{\;+\;L}_{S\_8\times 8}^{\prime }{\;+\;L}_{S\_16\times 16}^{\prime }{\;+\;L}_{S\_32\times 32}^{\prime }{\;+\;L}_{S\_64\times 64}^{\prime }{\;+\;L}_{S\_128\times 128}^{\prime }{\;+\;L}_{S\_256\times 256}^{\prime }$$

(15) $$L_{C\_\mathrm{totall}}{\;=\;L}_{C\_4\times 4}{\;+\;L}_{C\_8\times 8}{\;+\;L}_{C\_16\times 16}{\;+\;L}_{C\_32\times 32}{\;+\;L}_{C\_64\times 64}{\;+\;L}_{C\_128\times 128}{\;+\;L}_{C\_256\times 256}$$

The optimization goal of the discriminator D is to maximize $L_{S\_\mathrm{totall}}^{\prime }{\;+\;L}_{C\_\mathrm{totall}}$, and the optimization goal of the generator is to maximize $L_{S\_\mathrm{totall}}^{\prime }-L_{C\_\mathrm{totall}}$. During the training process, train the discriminator five times and then train the generator once. Finally, through continuous adversarial training, the network can adapt to the task of generating multiscale high-quality images.

### 2.2. Ship Detection Using Yolo v3 Model and Composite Dataset

The Yolo v3 [48,49,50,51,52,53] network was selected to test whether the SAR samples generated by our method can enhance the detection sample set.

Yolo v3 is an improvement based on Yolo v2, which is faster and more accurate. Its backbone network is darknet53. Yolo v3 will generate three different scale feature maps, which correspond to 1/32, 1/16 and 1/8 of the original size. Each scale corresponds to N channels, which contain the prediction information, the prediction results of each grid and the prior frames of each size. Yolo v3 uses the K-means clustering method to set three kinds of anchors for each subsampling scale, and nine kinds of anchors are clustered. Finally, the prediction frame is drawn on the predicted ship.

**Figure 3:**
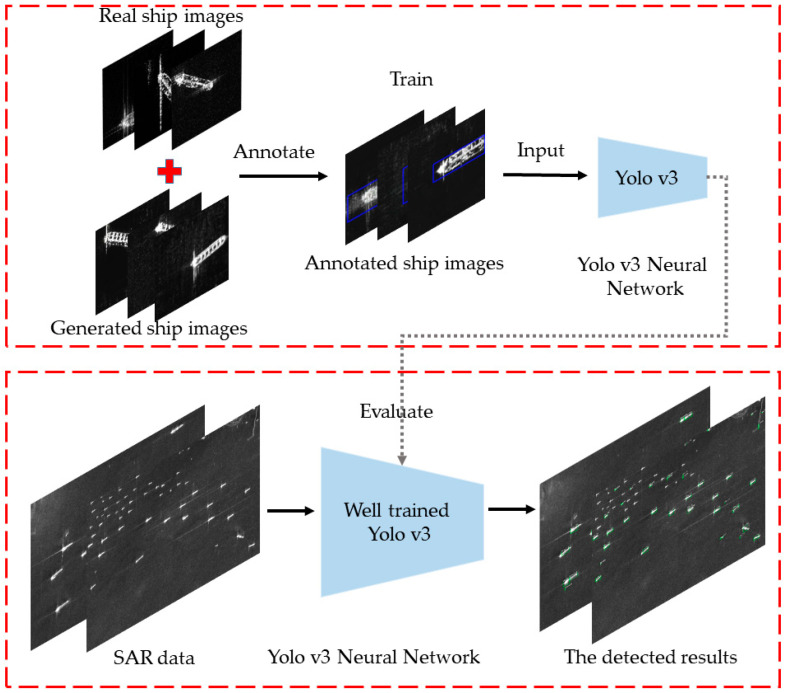
The flowchart of ship detection using Yolo v3 model and the composite dataset.

The flowchart of ship detection using the Yolo v3 model and the composite dataset is shown in Figure 3. The SAR image to be detected is segmented by a chessboard with the size of S × S, and then predict each sub image in SAR image by sliding window. Then, the sub images are resampled as the input size of the network, and input into the trained Yolo v3 network. Basic features are extracted from the backbone network, and multi-scale features can be extracted by fusing extra convolution layer features. Using the network calculation, the position parameters and labels of the prediction box on sub images are output. Finally, through coordinate conversion, the coordinates of the prediction frame are mapped to the original image, and the prediction results of all sub images are integrated and output.

## 3. Experiment

### 3.1. Data Set Description

**Figure 4:**
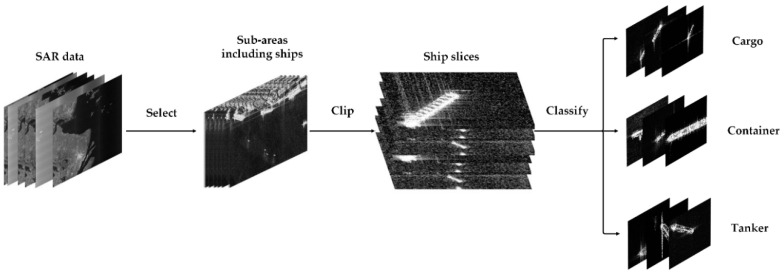
The process of making high-resolution Synthetic Aperture Radar (SAR) ship slices.

The Gaofen-3 (GF-3)3 m resolution SAR data are used in the experiment [54]. After obtaining the data, the radiometric calibration and normalization are carried out. Then, the “Imagelabel” tool is used to slice the original data; the process of making high-resolution SAR ship slices is shown in Figure 4. The slices include cargo ships, container ships, and tankers. The size of each slice is 256 × 256. The final number of slices is shown in Table 1.

### 3.2. Network Parameters Setting

All experiments use the Pytorch deep learning framework (version 1.5.1). The Anaconda3 virtual environment is used for management. The Python version is 3.7, the CPU is an Intel i7-8700 K, and the GPU is an NVIDIA GeForce GTX 1080 Ti. The kernel size of the generator and discriminator filters in the convolutional layer is set to 3. The upsampling process is realized by nearest neighbor interpolation. The optimizer is “Adam”, the learning rate is 0.001, and beta1 and beta2 are set to 0.9 and 0.999, respectively.

During Yolo v3 training, the batch size is set to 64, the learning rate is set to 0.001, and the Adam optimizer is used. The network input pixel is set to 256 × 256. The threshold of intersection over union (IOU) is set to 0.5, the thresholds for the target confidence range are set to 0.3 and 0.5, and the number of iterations is 10,000.

### 3.3. Experimental Results

**Figure 5:**
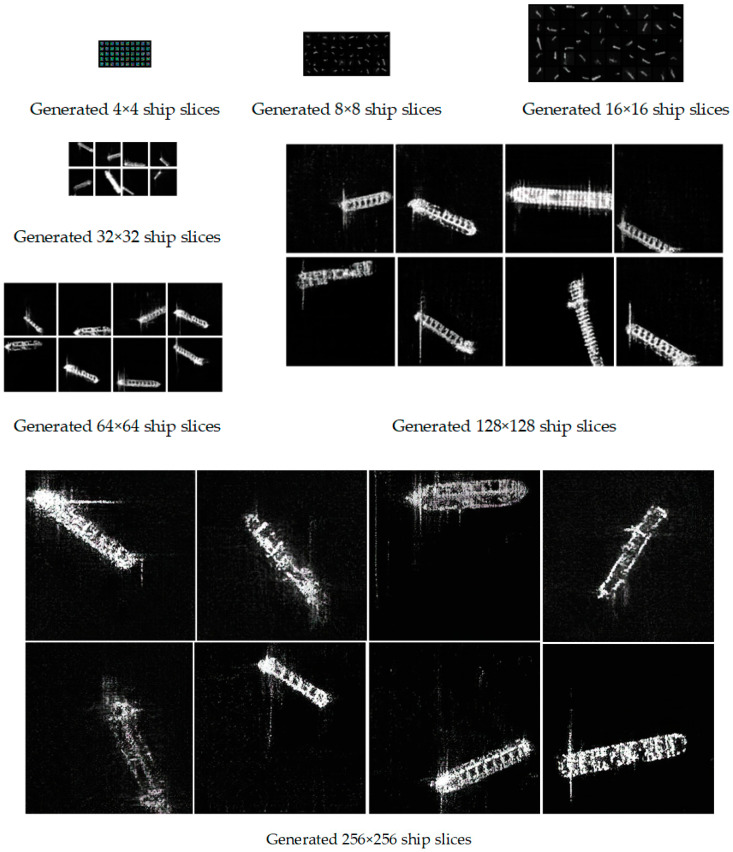
Different scales of SAR ship slices generated by the Multiscale Wasserstein Auxiliary Classifier Generative Adversarial Networks (MW-ACGAN).

Based on the real ship dataset, 4 × 4 to 256 × 256 high resolution SAR ship slices are generated by our proposed MW-ACGAN method, as shown in Figure 5. It can be seen from the results in the figure that the MW-ACGAN network has preliminarily realized the learning of three types of ship structures, which can simulate and characterize the unique image features of SAR images. The experimental results also show that in the process of generating 4 × 4 ~ 256 × 256 ship slices by MW-ACGAN, the image clarity and resolution are gradually improved, and the hull contour and texture are more realistic. It shows that with the increase in the number of network model layers, the image clarity is gradually improved, and the fitting ability of the generator to the real ship SAR slice is enhanced.

**Figure 6:**
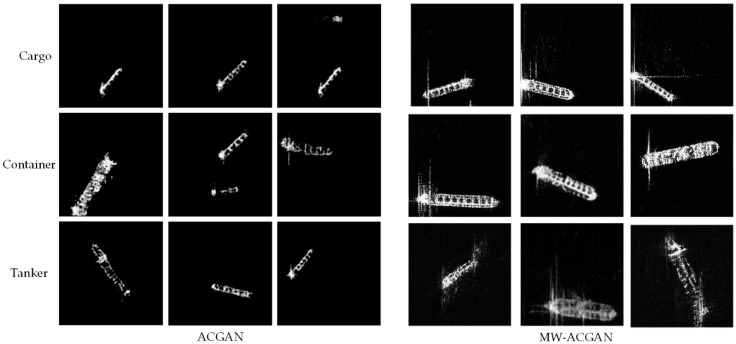
Comparison of ship slices generated by the ACGAN and the MW-ACGAN.

Figure 6 shows ship slices generated by the ACGAN and the MW-ACGAN, respectively. Obviously, the two models can generate different kinds of ship slices and have good contour information. However, compared with MW-ACGAN model, ACGAN has obvious shortcomings. Some generated ship slices have certain fuzziness, and their texture features and edge features are also relatively fuzzy. The scattering sidelobe and speckle of the original ship slice are lost. In addition, due to the lack of network fitting ability, some generated ship slices appear with a curve shape, resulting in image distortion.

**Figure 7:**
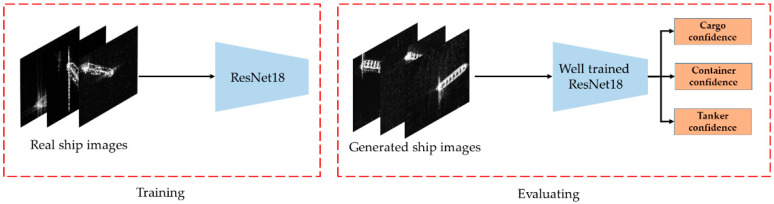
The Generative Adversarial Networks (GAN) test evaluation method based on ResNet18 network.

From Figure 5 and Figure 6, we can see that our method can generate ship slices of different sizes and categories. In order to further analyze whether the ship slices generated by the MW-ACGAN have species diversity, we use the GAN test [55] method to evaluate he quality of the generated images. ResNet18 [56] is used as the benchmark classification network, as shown in Figure 7. Here, we change the last output layer of the original ResNet18 from 1000 categories to 3 categories to ensure consistency with the SAR ship data set. The image size is 256 × 256, the learning rate is 0.008, the batch size is 32, the Adam momentum optimizer is used, the betas are set to 0.5 and 0.99, and the loss function is CrossEntropyLoss. The final iteration epoch is set to 1000.

Firstly, the ResNet18 network is trained with the real 256 × 256 SAR ship data. The number of training set and test set is shown in Table 2. Then, the score of ResNet18 network is used as a quantitative evaluation method to generate SAR ship images. When the score level of the generated image is higher, the quality of the generated SAR image is higher, and the similarity with the real SAR image is greater.

The ACGAN network and MW-ACGAN network are used to generate 1000 slices of cargo ships, container ships and oil tankers. Then, the slices are scored with the trained Rensnet18. Table 3 shows the ResNet18 scores for three different types of ships. The ResNet18 scores of MW-ACGAN in all ship categories are better than those of ACGAN, and the average score is better than 0.9. This shows that the method proposed in this paper can better understand the characteristics of ships and make ship slices more realistic.

To verify that the generated high-resolution ships are still practical for target detection scenarios, In order to verify the practicability of the high-resolution ship detection scene, Yolo v3 model is used to carry out ship detection experiments in SAR images.

Using the original ship set, the original ship set plus 2000 MW-ACGAN slices, and the original ship set plus 4000 MW-ACGAN slices as the training and test data, the Yolo v3 model is trained, respectively, to form three trained Yolo v3 models, and then the trained Yolo v3 models are tested on three real SAR images. In the whole training process, the number of iterations of different experiments remains unchanged.

**Figure 8:**
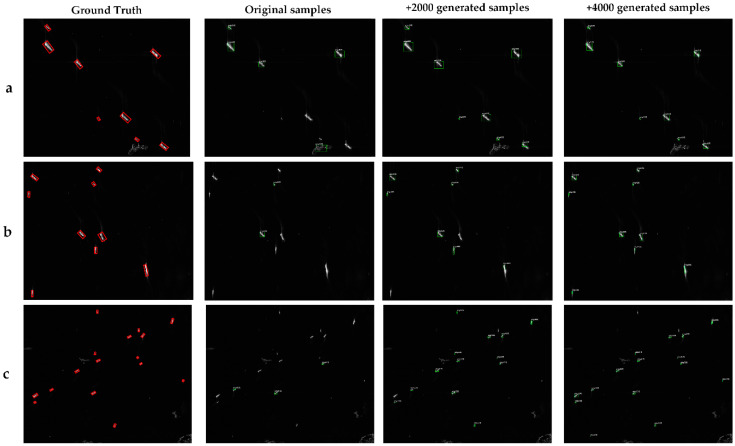
Detection results of (**a**,**b**,**c**) SAR images using Yolo v3 model trained by different sample sets.

Figure 8 and Table 4 show the detection results of three SAR images using the Yolo v3 model trained by the three sample sets, here the target confidence is set to 0.3. In Table 4, the Yolo v3 model after training in the original data set only detects two ships in the b SAR image. When 2000 and 4000 MW-ACGAN generated slices are added, 7 and 9 ships are detected. As expected, the use of the GAN to generate enhanced ship data can effectively increase the detection rate. The more images you add, the more the detection rate increases.

Table 5 shows the statistics of Yolo v3 detection results. Using only the original training data, the detection rate of Yolo v3 is only 27% when the target confidence is 0.3. This may be due to the fact that the original samples are too small to overfit the model after 10,000 iterations. When 2000 generated slices are added, the detection rate is increased by 58%, which indicates that the number of ship modes in the data set is increased and the generalization ability of the model is improved. The detection rate reaches 94% when 4000 slices are added to the original data set, which indicates that the ship image generated by MW-ACGAN has high quality and multiclass features, which can effectively improve the generalization ability of the detection model and play an important role in the detection scenarios.

## 4. Conclusions

In order to meet the application requirements of ship detection in high-resolution SAR images with small samples, we propose a ship detection method based on the SAR image generation network (MW-ACGAN) and the detection network Yolo v3 under small samples. The experimental results of GF-3 SAR data show that the MW-ACGAN network can effectively generate realistic multiclass ship images by using the Wasserstein distance and gradient penalty. The multi-scale loss term can effectively generate multi-scale ship images. The generated ship slices show good class separability under ResNet18 classifier, which can also be applied to the study of ship classification in the future. In the process of Yolo v3 network detection, the original small sample SAR data set is effectively expanded by using the generated samples, and the detection accuracy of three SAR images is better than 90%, which proves the feasibility of our method.

In the future, we will further study the generation of SAR ship images under different sea conditions, different polarization modes, and different incident angles. We will combine deep learning abstract features and traditional concrete ones to further improve the accuracy of the generated features. In the next step, more systematic experimental analysis will be carried out, and generated images will be applied to ship detection and classification under multiple scenes and complex sea conditions, so as to improve the detection rate of ships in complex scenarios.

## Figures and Tables

**Table 1:** Gaofen-3 (GF-3) high resolution ship slices.

Ship	Cargo	Container	Tanker	Total
Number	342	262	231	835

**Table 2:** ResNet18 training set and test set.

Ship Types	$S_{\mathrm{train}}$	$S_{\mathrm{test}}$
Cargo	279	63
Container	211	51
Tanker	185	46
Total number	675	160

**Table 3:** ResNet18 scores of the ACGAN and the MW-ACGAN.

Category	ACGAN’s Average Score	MW-ACGAN’s Average Score
Cargo	0.91	0.93
Container	0.78	0.88
Tanker	0.85	0.92
Average	0.85	0.91

**Table 4:** Statistics of Yolo v3 detection results.

Image ID		Object Confidence >= 0.3						Object Confidence >= 0.5					
	Total Number of Ships	835 Slices		+2000 Slices		+4000 Slices		835 Slices		+2000 Slices		+4000 Slices	
		Detec-Ted	FALSE ALARM	Detec-Ted	False Alarm	Detec-Ted	False Alarm	DETEC-TED	False Alarm	Detec-Ted	False Alarm	Detec-Ted	False Alarm
a	8	4	1	8	0	8	0	2	0	5	0	8	0
b	9	2	0	7	0	9	0	2	0	3	0	7	0
c	16	3	0	13	0	15	0	2	0	8	0	8	0

**Table 5:** Statistics of Yolo v3 detection results.

	Object Confidence ≥ 0.3		Object Confidence ≥ 0.5	
	Accuracy	False Alarm Rate	Accuracy	False Alarm Rate
835 real ship slices	27%	3%	18%	0
+2000 MW-ACGAN generated slices	85%	0	48%	0
+4000 MW-ACGAN generated slices	94%	0	70	0
